# SBML-SAT: a systems biology markup language (SBML) based sensitivity analysis tool

**DOI:** 10.1186/1471-2105-9-342

**Published:** 2008-08-15

**Authors:** Zhike Zi, Yanan Zheng, Ann E Rundell, Edda Klipp

**Affiliations:** 1 Computational Systems Biology, Max Planck Institute for Molecular Genetics, Ihnestr. 73, 14195 Berlin, Germany; 2 Entelos Inc., 110 Marsh Drive, Foster City, CA 94404, USA; 3 Weldon School of Biomedical Engineering, Purdue University, West Lafayette, IN 47907, USA; 4 Theoretical Biophysics, Humboldt University Berlin, Institute for Biology, Invalidenstr, 42, 10115 Berlin, Germany

## Abstract

**Background:**

It has long been recognized that sensitivity analysis plays a key role in modeling and analyzing cellular and biochemical processes. Systems biology markup language (SBML) has become a well-known platform for coding and sharing mathematical models of such processes. However, current SBML compatible software tools are limited in their ability to perform global sensitivity analyses of these models.

**Results:**

This work introduces a freely downloadable, software package, SBML-SAT, which implements algorithms for simulation, steady state analysis, robustness analysis and local and global sensitivity analysis for SBML models. This software tool extends current capabilities through its execution of global sensitivity analyses using multi-parametric sensitivity analysis, partial rank correlation coefficient, SOBOL's method, and weighted average of local sensitivity analyses in addition to its ability to handle systems with discontinuous events and intuitive graphical user interface.

**Conclusion:**

SBML-SAT provides the community of systems biologists a new tool for the analysis of their SBML models of biochemical and cellular processes.

## Background

With growing interest in systems biology, mathematical models have been widely used to study metabolic networks, gene regulatory networks and cell signaling pathways [1-6]. These mathematical models are used to reproduce experimental data and predict unobserved behaviors of the system. However, many sources of uncertainty including errors, inconsistency and noise of experimental data, absence of parameter information, incomplete representation of underlying process details, and poor understanding of the biological mechanisms impose a limit on model confidence. Furthermore, intrinsic variability or noise of the system such as the occurrence of stochastic events also affects the output of the model. Therefore, it is important not only to understand the dynamical properties of the model with particular parameter values, but also to further investigate the effect of their perturbations on the system [7]. Sensitivity analysis is a powerful approach for investigating which parameters in a model have the strongest effect on overall behavior. In addition to identifying key parameters in a model, sensitivity analysis is valuable in pinpointing parameters, which should be in the focus of experimental perturbation [8].

Sensitivity analysis has been widely utilized for the systems biology research [2,7,9-16]. However, it is time consuming for researchers to apply different algorithms to their specific models. In order to automate sensitivity analysis for different types of systems biology models, we developed a free software tool named SBML-SAT: a systems biology markup language (SBML) based sensitivity analysis tool. SBML is a language developed by the systems biology community to represent and exchange models of biochemical reaction networks [17]. SBML is being used by a large group of software developers and researchers. More than 120 software systems have so far been developed for supporting SBML . Although a few existing software systems such as COPASI [18] and SBToolbox [19] incorporate local sensitivity analysis, a powerful, flexible and broadly applicable sensitivity analysis platform is still lacking. In particular, some important features missing from the existing software systems are described below.

Firstly, some mathematical models of biological system include discontinuous events, such as the division of cells, removal of biological signal at a specific time or blocking protein synthesis during an experiment. Most existing SBML supported software systems (except for SBML-PET [20], MathSBML [21], SBTOOLBOX2 [19], etc.) do not support models involving such discontinuous events. The broad applicability of these software systems is thus limited.

Secondly, none of the existing SBML software packages allows for global sensitivity analysis. A few of the existing software systems can run local sensitivity analysis which introduces a small perturbation of one parameter for each simulation. Therefore, local sensitivity analysis investigates sensitivity of the model outputs with respect to a particular point in the parameter space. However, a single "true" point of parameter set may not occur in nature. It is likely that biological parameters such as rate constants and initial concentrations are variable in a large range depending on the specific cell types and cellular environments. For this reason, a global sensitivity analysis is valuable to explore sensitivities of model outputs to simultaneous variations of all the parameters over a large range and examine possible non-linear effects of the parameters as well as their interactions [7].

Thirdly, the results of sensitivity analysis correspond to specific model outputs. The specific model outputs of interest usually vary from case to case. In some cases, users may want to study the integrated or maximum response of certain species, while in other cases interest may be placed on particular time dependent or steady state responses of the system. Thus, a good sensitivity analysis software platform should provide various options for specifying model outputs.

Here, we present the software system SBML-SAT that encompasses all of the above capabilities. It is worth pointing out that the purpose of this paper is not to explain the technical details of the software (described in the manual file) or the published algorithms, nor to present any particular biological findings. Rather, we provide an overview of the software, its validation with a variety of mathematical models for biological systems and demonstrate its broad applicability.

## Implementation

### Overview of the software system

SBML-SAT is designed to run simulation, steady state analysis, robustness analysis, as well as local and global sensitivity analysis for ordinary differential equations (ODE) based biological models. SBML-SAT meets the needs mentioned in the rational section with the following features:

Inspired by our previous work in SBML-PET project [20], we enabled SBML-SAT to support a variety of models including assignment rules and events, even for complicated event scenarios such as bisecting mass in case of cell division. Therefore, SBML-SAT will have a wide applicability for different types of models.

In addition to the implementation of traditional local sensitivity analysis, SBML-SAT provides four different global sensitivity analysis methods, including multi-parametric sensitivity analysis [7,12], partial rank correlation coefficient analysis [9,22], SOBOL's method [15,23] and weighted average of local sensitivities [2]. Furthermore, steady state analysis and robustness analysis are also available in this tool. The algorithms for these different types of analyses are briefly described in the following section.

The sensitivity analysis can be performed with respect to any ODE model variable (species amount or concentration) and reaction rate; these quantities are referred to as the object of the sensitivity analysis in SBML-SAT. The model output, which the sensitivity analysis is performed on, is defined through a functional operation on the object. The predefined model outputs in SBML-SAT are: steady state response, maximum response, integrated response, and time dependent response. The steady state response is only applicable for model objects that are ODE variables as the sensitivity analysis is computed with respect to the equilibrium solution of the system (when all derivatives of the ODE variables are algebraically set to zero). The maximum response is the maximum value of the object *X*_*i *_(state variable or reaction rate) over the time course simulated:

(1) $$\underset{t\in \left\{t_{0},\;t_{1},\;...,\;t_{end}\right\}}{\text{max}}\left[X_{i}\right]\left(t\right)$$

The integrated response corresponds to the area under the curve when plotting *X*_*i *_versus time. SBML-SAT approximates the integrated response for object *X*_*i *_by the discrete summation [24]:

(2) $$I_{i}=\int_{t_{0}}^{t_{end}}\left[X_{i}\right]dt≌\frac{∆t}{2}\left(\left[X_{i}\right]\left(t_{0}\right)+2\left[X_{i}\right]\left(t_{1}\right)+....+2\left[X_{i}\right]\left(t_{n-1}\right)+\left[X_{i}\right]\left(t_{n}\right)\right)$$

The time dependent response performs multiple sensitivity analyses based on the values of the object, *X*_*i*_, at selected time points during the simulated time course.

SBML-SAT for Windows, Mac, and Linux can be freely downloaded from its website . The manual documentation file including a detailed tutorial for the usage of SBML-SAT is also available in the website. The future updates of SBML-SAT will be released on the website as well. Like most other SBML supported software systems, SBML-SAT requires a link to libSBML and utilizes SBMLToolbox [25], which allows us to import SBML into MATLAB [26]. Once the SBML model is imported into SBML-SAT, a MATLAB file will be automatically generated, which includes the ODEs of the model. This is very helpful for the user, who wants to code in MATLAB for other purposes. To speed up the process of solving the ODEs, we employed the CVODE module of SUNDIALS (Suite of Nonlinear and Differential/Algebraic Equation Solvers) as the ODE Solver [27]. An interface to setting the options of CVODE solver is also available in SBML-SAT. Both SBMLToolbox and SUNDIALS [28] can be freely downloaded.

In order to run the analysis in SBML-SAT, the users need to represent their models in SBML format which can be easily done using the existing software systems such as CellDesigner [29], COPASI [18] and SBMLeditor [30]. Then, the users can load the SBML models to SBML-SAT and perform a variety of analyses.

**Figure 1:**
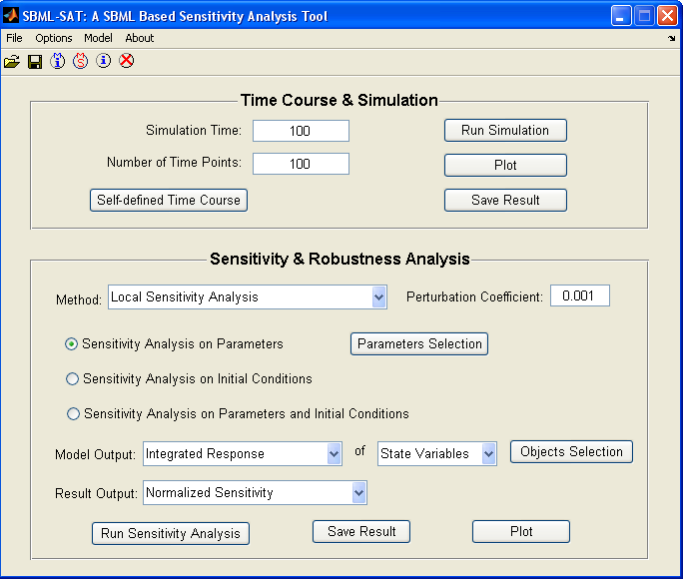
**GUI of SBML-SAT**. The graphic user interface (GUI) of SBML-SAT provides an easy way for the user to run the simulation, steady state analysis, sensitivity analysis and robustness analysis for SBML models.

Simulation, robustness analysis and sensitivity analysis can be easily implemented using SBML-SAT's graphical user interface (Figure 1). SBML-SAT allows the user to browse the model information, to save the model as well as to simulate and analyze the model. Simulation and sensitivity analysis results can be exported as text files, making it convenient for post-processing. In addition to the export function, SBML-SAT provides automatic visualization of the results. Furthermore, SBML-SAT is smart in remembering the user's settings for the corresponding tasks. The user can save his/her project settings as a project file and load it again to SBML-SAT for further analysis later.

### Local sensitivity analysis

Local sensitivity analysis is a study of the changes in the model outputs with respect to parameter (factor) variations around a local point in the parameter space, which are quantified by the sensitivity coefficients. Mathematically, the sensitivity coefficients are the first order derivatives of model outputs with respect to the model parameters:

(3) $$S_{ij}=\frac{\partial O_{i}}{\partial p_{j}}$$

where *O*_*i *_is the *i*-th model output and *p*_*j *_is the *j*-th parameter. This is called "Unnormalized Sensitivity" in SBML-SAT. SBML-SAT employed centered difference approximation to compute the sensitivity coefficients in the following way [31]:

(4) $$S_{ij}=\frac{\partial O_{i}}{\partial p_{j}}\approx \frac{O_{i}(p_{j}+∆p_{j})-O_{i}(p_{j}-∆p_{j})}{2∆p_{j}}.$$

When the model output and parameters are non-zero, the normalized sensitivity coefficients are defined as:

(5) $$S_{ij}^{normalized}=\;\frac{\;\frac{\partial O_{i}}{O_{i}}\;}{\frac{\partial p_{j}}{p_{j}}}\approx \frac{\;\frac{O_{i}(p_{j}+∆p_{j})-O_{i}(p_{j}-∆p_{j})}{O_{i}}\;}{\frac{2∆p_{j}}{p_{j}}}.$$

The new model outputs are calculated by a small perturbation (Δ*p*_*j*_) of parameter *p*_*j *_while keeping all the other parameter values the same: SBML-SAT computes one-at-a-time (OAT) local sensitivity coefficients.

The proper choice of perturbation size is a delicate issue as it depends on the nature of the model and the numerical solution method. The perturbation should be small enough to achieve a negligible error in the centered difference approximation, and large enough to be unaffected by the numerical inaccuracies of the ODE solver. Too large parameter perturbation violates the implied linearity of the approximations in (4) and (5) and will provide inaccurate results. The user can modify the perturbation coefficient in the "Sensitivity Analysis" panel of SBML-SAT. The default perturbation is 0.1% of the corresponding parameter value, ie. Δ*p*_*j *_= 0.001 × *p*_*j*_.

### Global sensitivity analysis

As mentioned in the rationale section, there are many sources of uncertainty in the model parameter values. Global sensitivity analysis is a useful way to investigate the global effects of parameters on the model output by simultaneously perturbing all the parameters within a parameter space. In the SBML-SAT tool, four different global sensitivity analysis methods are available. Each method has a distinct mathematical rationale and can be used for different purposes.

(1) Multi-Parametric Sensitivity Analysis (MPSA): This method was first proposed by Hornberger et al [32] in the field of hydrology and further applied to modeling of biological systems by Cho et al. [12] and Zi et al. [7]. MPSA can be used to study the relative importance of the parameters with respect to the model output. The basic idea of MPSA is to map the uncertainty of the parameters into the model output by randomly generating parameter values from predefined distributions (without prior knowledge, uniform distributions are assumed). SBML-SAT uses Latin Hypercube Sampling (LHS) method to sample the parameter values under the given ranges of the parameters [7]. The LHS method is an efficient method to sample random parameter vectors while guaranteeing that individual parameter ranges are evenly covered [33]. The ranges of the parameter distributions are usually determined from the available literature or guided by experience of the researchers.

For each randomly generated parameter set, the objective function is computed by the sum of square errors between the model outputs from the random parameter set and the reference parameter set. The next step is to classify each parameter set as acceptable or unacceptable by comparing its objective function value to the average of all the objective function values. If the objective function value is smaller than the average, the parameter set is classified as "acceptable"; otherwise it is "unacceptable". Then, the cumulative frequency is calculated for both acceptable and unacceptable cases for each selected parameter with increasing parameter values. Finally, the sensitivity of the parameter is measured by the maximum vertical distance of the two cumulative frequency curves according to the Kolmogorov-Smirnov statistics [7]. The calculated MPSA sensitivities are between 0 and 1, where a value closer to 1 indicates a relatively higher importance of the parameter variation to the overall corresponding model output.

(2) Partial Rank Correlation Coefficient Analysis (PRCC): The PRCC method is a rank transformed linear regression analysis that is routinely used for analysis of systems with a nonlinear and monotonic relationship between the system inputs and outputs [22]. Linear regression analysis methods best fit a straight line to input and output values. When nonlinear, monotonic relationships exist between system input and outputs, poor linear regression fits can be alleviated by performing the linear regression analysis on a rank ordered list of the model output and input values. PRCC calculates the sensitivity indices from the Pearson correlation coefficients between the model output and input parameters as well as each pair of parameters after rank transformation [9]. Interactions among different parameters are eliminated by evaluating multiple regression models on a subset of parameters that excludes a single parameter. The calculated PRCC sensitivity indices are a standardized sensitivity measurement between -1 and 1 with 0 indicating an input to which the model output is completely insensitive. SBML-SAT computes PRCC as implemented in [15] with LHS sampling of the parameter space.

(3) SOBOL's Method: SOBOL's method is a variance based method that makes no assumptions on the relationship between the system inputs and outputs. It is computationally expensive since it utilizes a large number of model simulations with parameter values sampled from the parameter space by the winding stair algorithm. The variance of the numerous model outputs is estimated by Monte Carlo integrations. The model output variance is apportioned into summands of partial variances from combinations of input parameters with increasing dimensionality [23]. The total effects sensitivity indices quantify all of the effects that a parameter, in combination with any other parameter(s), has on the model output. They are defined as the ratio of the sum of the related partial variances to the overall variance of the model output. The larger the fraction, the higher is the corresponding sensitivity. SBML-SAT calculates the total effect sensitivity indices.

(4) Weighted Average of Local Sensitivities: In this approach, local sensitivity indices are calculated at multiple random points within the parameter space; a weighted average of the local sensitivity indices serves to provide some approximation of the global parameter sensitivities. Bentele et al. [2] proposed a Boltzmann-Distribution weighting function, exp(-E/k_b_T), where E is the error between the model simulation and experimental data and k_b_T is a customizable scaling factor. Herein we define E as the least squares error (LSE) between the perturbed model simulation and reference model simulation and k_b_T as the minimum LSE. Based on this weighting function, the random points in the parameter space with low LSE contribute the most to the calculated global sensitivity indices.

### Steady state analysis

SBML-SAT uses two different methods to check the existence of a steady state for the SBML model. The first strategy is to set the ordinary differential equations to zero and solve the algebraic system by KINSOL, which is part of the software family called SUNDIALS and is an algebraic system solver based on Newton-Krylov method [27]. Another method is called quasi steady state method, which runs the simulation for a very long time and check the rate of change of the ODE variables (such as species and other state variables) at different time points. When the rates of change for all the variables are smaller than a certain threshold (1 × 10^-10^), a quasi steady state is reached. The latter method is useful for steady state analysis of models that include events and implicit mass conservation rules. These two methods will only find a single steady state to which the initial condition converges. Other existing steady states as well as the steady state of oscillatory and unstable system will not be detected. SBML-SAT automatically selects the method for steady state analysis. If the model doesn't have events, SBML-SAT will use the algebraic method to detect the steady state of the model. Otherwise the second quasi steady state method will be used.

### Robustness analysis

Robustness is one of the fundamental properties of biological systems, which allows the system to maintain its behavior against random perturbations [34,35]. SBML-SAT employs a method proposed in previous studies to investigate the robustness of model output against the total parameter variation, *TPV*, which is defined as [36-38]:

(6) $$TPV=\sum_{n=1}^{L}\left|\log_{10}\left(\frac{k_{n}}{k_{n}^{0}}\right)\right|$$

where *k*_*n *_are the perturbed model parameters randomly generated by the LHS method; $k_{n}^{0}$ are the corresponding reference parameter values in the model; *L *is the total number of parameters that are randomly varied.

To measure robustness, we use the robustness metric $R_{output,TPV}^{M}$, which quantifies the change in a function of the system (model output) induced by TPV:

(7) $$R_{output,TPV}^{M}=\frac{-\sum_{p=1}^{N}\left|\log_{10}\left(\frac{f_{p}}{f_{0}}\right)\right|}{N}.$$

where *f*_0 _and *f*_*p *_are the model output which describes the biological function under non-perturbed condition (reference model) and perturbed condition (parameters varied model), respectively. *N *is the total number of perturbations or model simulations. *M *denotes the model for the corresponding system. When the reference model output is zero, the following alternative definition is used:

(8) $$R_{output,TPV}^{M}=\frac{-\sum_{p=1}^{N}{\left(f_{p}-f_{0}\right)}^{2}}{N}.$$

According to the definition of (7) and (8), the robustness score of a biological system (model) usually assumes a negative value. The closer it is to zero, the more robust the system (model) is against the perturbations (parameter variations). When the robustness score of a system is zero, it means this system is absolutely robust against the imposed perturbations.

The difference between the robustness scores of two systems (models) with respect to a certain model output against the perturbations can be evaluated as:

(9) $$∆R_{output,TPV}^{M1,M2}=R_{output,TPV}^{M1}-R_{output,TPV}^{M2}.$$

The comparison of the robustness scores of two systems/models is meaningful only when the evaluated model output of the two systems/models are the same and perturbations are operated in the same way.

## Results

In this section, we will demonstrate the functions and broad applicability of SBML-SAT using a variety of mathematical models for the biological systems. All of the models presented here are pre-encoded in SBML format and most of them are taken from the BioModels Database [39]. At the start of each subsection, a brief description of the instructions to operate SBML-SAT for each function are provided to enable the reader to further envision the interaction with the software tool and facilitate its use.

### Simulation of SBML models

To simulate a pre-constructed SBML model, the user loads the SBML model, sets the time course for simulation, and selects "Run Simulation".

**Figure 2:**
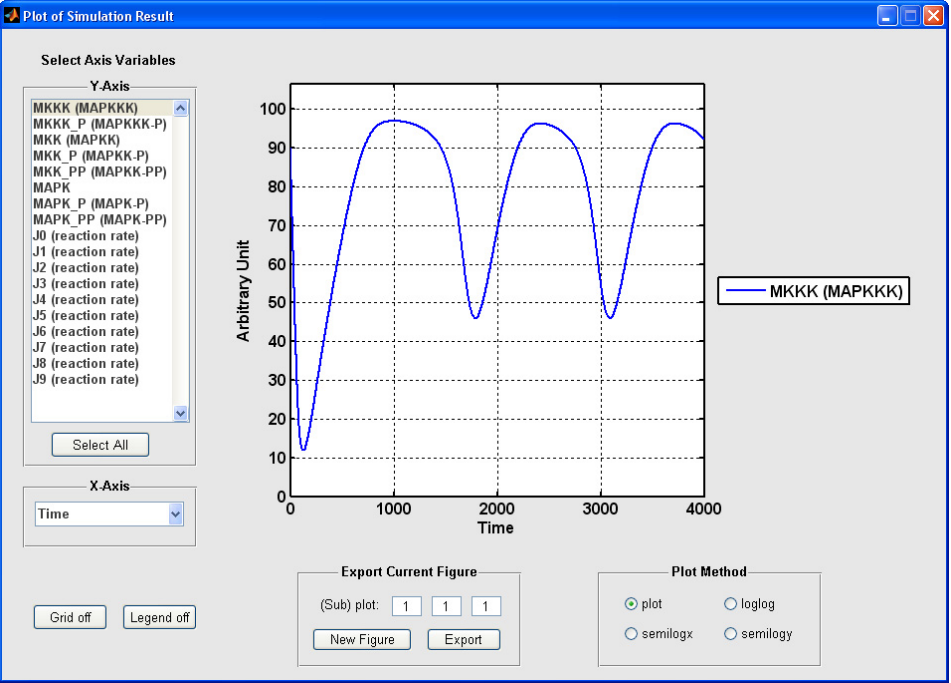
**Plot of simulation result in SBML-SAT**. The plot function enables the user to visualize the time course profiles of species and reaction rates. This graph shows the simulation result of the MAPK cascade model [43] (BioModels ID: BIOMD0000000010).

**Figure 3:**
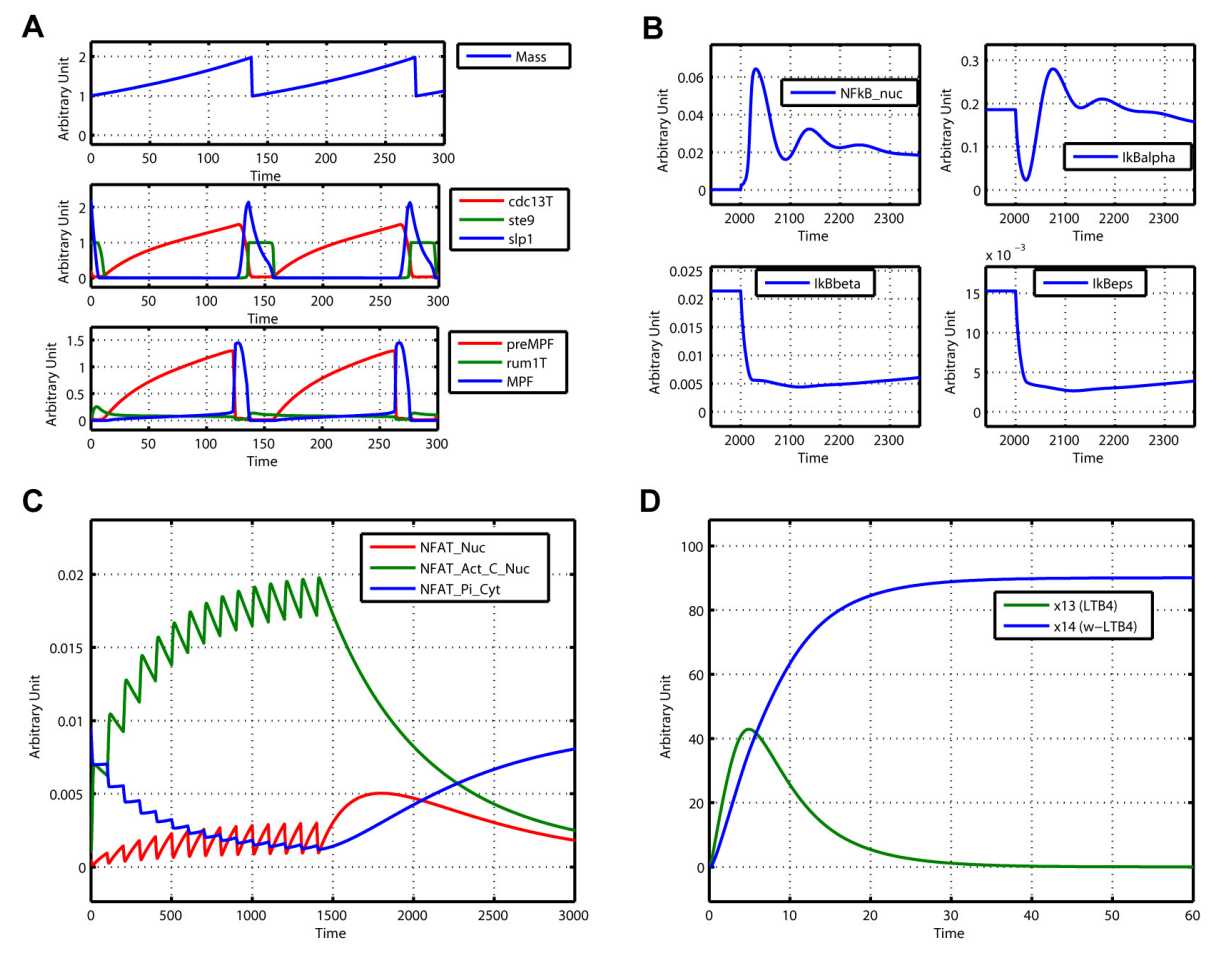
**Simulation of different types of models in SBML-SAT**. (A) Simulation result of the fission yeast cell cycle model (events included, BioModels ID: BIOMD0000000111), identical to Fig. 4 of [44]. (B) Simulation result of a NF-κB signalling pathway model (BioModels ID: BIOMD0000000140), identifical to Fig. 2F of [1]. (C) Simulation result of a T cell gene expression model (BioModels ID: BIOMD0000000122), identical to Fig. 4a of [45]. (D) Simulation result of a metabolic model (BioModels ID: BIOMD0000000106), identical to Fig. 2A of [46].

SBML-SAT provides an easy way to run a simulation and visualize the simulation results for SBML models. The output screen for SBML-SAT model simulation is shown in Figure 2. In order to test the wide applicability of SBML-SAT, we ran simulations for a variety of models from the BioModels Database, which include biophysical models, signaling pathways, gene expression and metabolic networks. The results shown in Figure 3 demonstrates that SBML-SAT appropriately simulates both continuous SBML models (signaling pathway, gene expression and metabolic models), as well as those with discontinuous events (cell cycle model) with different degrees of complexity and nonlinearity.

### Sample local sensitivity analysis

To conduct the local sensitivity analysis, the user

• loads the SBML model,

• sets the time course,

• chooses the parameter(s),

• defines the perturbation coefficient, and

• selects the objects (ODE variables or reaction rates) and the model output operation for the analysis,

• select the appropriate analysis approach to run.

**Figure 4:**
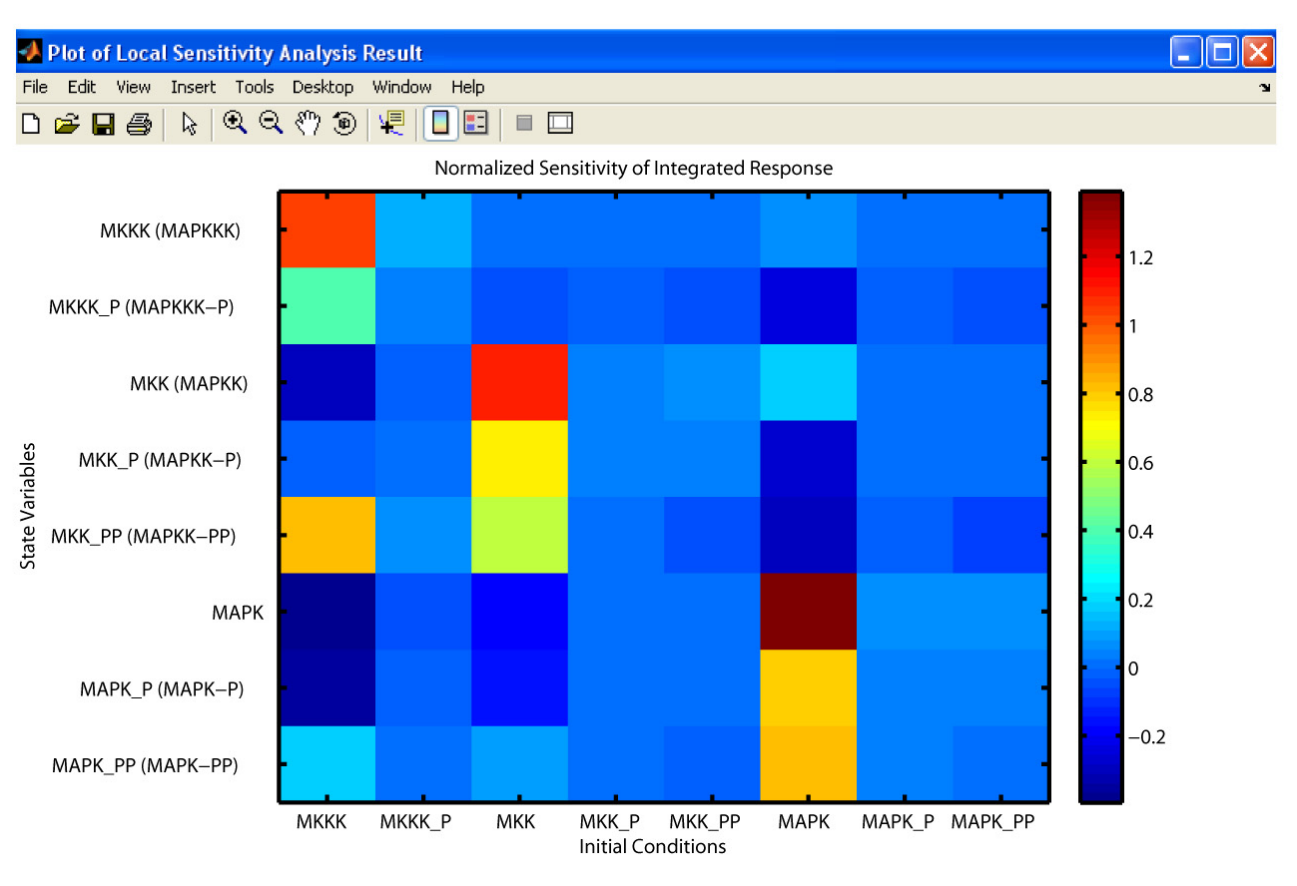
**Local sensitivity analysis in SBML-SAT**. Local sensitivity analysis of the integrated response of MAPK cascade model [43] (BioModels ID: BIOMD0000000010) with respect to variation of initial conditions.

The result of a SBML-SAT normalized local sensitivity analysis on the MAPK cascade model (BioModels ID: BIOMD0000000010) is shown in Figure 4. For this analysis, the objects of the sensitivity analysis were the state variables associated with the various phosphorylated forms of the MAPK cascade elements and the model output analyzed were the integrated responses. The parameters perturbed were the initial concentrations of each form with the default perturbation coefficient. These results indicate that the integrated response of the MAPK concentration was the most sensitive to the initial concentration of MAPK.

### Sample global sensitivity analysis

The user interface and operation for performing global sensitivity analyses is similar to that for the local sensitivity analysis: the user specifies the time course, object(s) and parameter(s) as well as the model output(s) for global sensitivity analysis. In addition, the user chooses the global sensitivity analysis method, and sets the variation range of the parameter values. The user must also define the number of Monte-Carlo simulations to be performed to base the analysis upon: this is highly dependent upon the nature of the model, the number of parameters (factors) to be analyzed, and the size of the parameter space (factor levels). The user needs to try different "Number of Simulations". If the analysis results are not significantly changed by the increasing of "Number of Simulations", then the results are assumed to be reliable and accurate enough. Once all these settings are done, SBML-SAT is ready to perform the specified global sensitivity analysis. The time required to complete the analysis varies from several minutes to several hours. It depends on the complexity of the model and the number of Monte-Carlo simulations.

**Figure 5:**
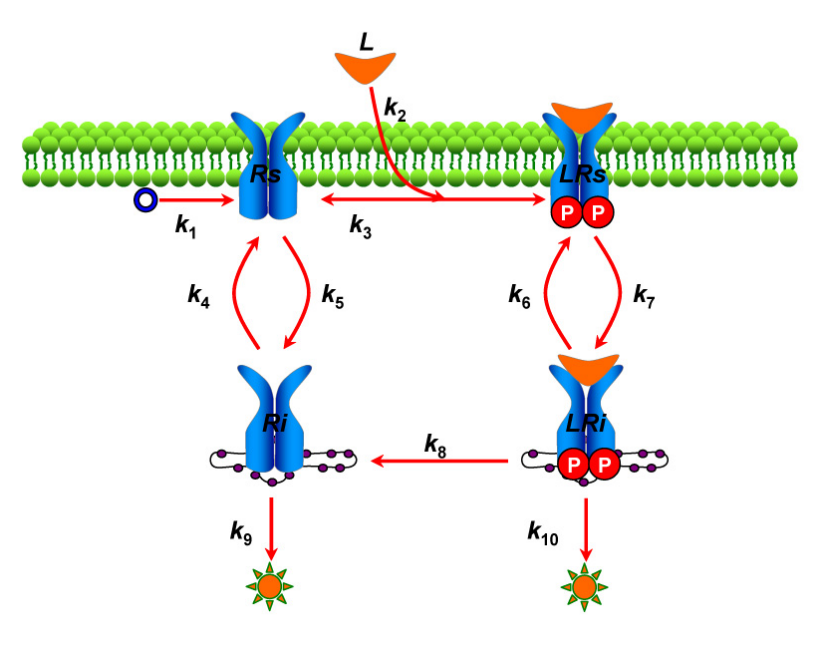
**Scheme of receptor trafficking network model**. Schematic description of the receptor trafficking network. The symbols *L, Rs, LRs, Ri, LRi *represent the ligand, unbound cell surface receptor, cell surface ligand-receptor complex, internalized unbound receptor and internalized ligand-receptor complex, respectively. The parameter information is listed in Table 1.

We use a model of the receptor trafficking network to demonstrate how to implement global sensitivity, steady state and robustness analyses in SBML-SAT. The general model of receptor trafficking networks is composed of the de novo production of surface receptor, ligand-receptor interaction, internalization, recycling and degradation of both empty and occupied receptors (Figure 5). The symbols of the parameters in the model and their corresponding biological processes are listed in Table 1. Detailed information about this model is available in our previous work [24,40].

**Table 1:** Parameters for the model of the receptor trafficking network

Symbols of Parameters	Corresponding Biological Processes
k_1_	de novo synthesis of surface receptor
k_2_	formation of ligand-receptor complex
k_3_	dissociation of ligand-receptor complex
k_4_	recycling of internalized unbound receptor
k_5_	internalization of unbound surface receptor
k_6_	recycling of internalized ligand-receptor complex
k_7_	internalization of surface ligand-receptor complex
k_8_	dephosphorylation of ligand-receptor complex
k_9_	degradation of unbound receptor
k_10_	degradation of ligand-receptor complex

**Figure 6:**
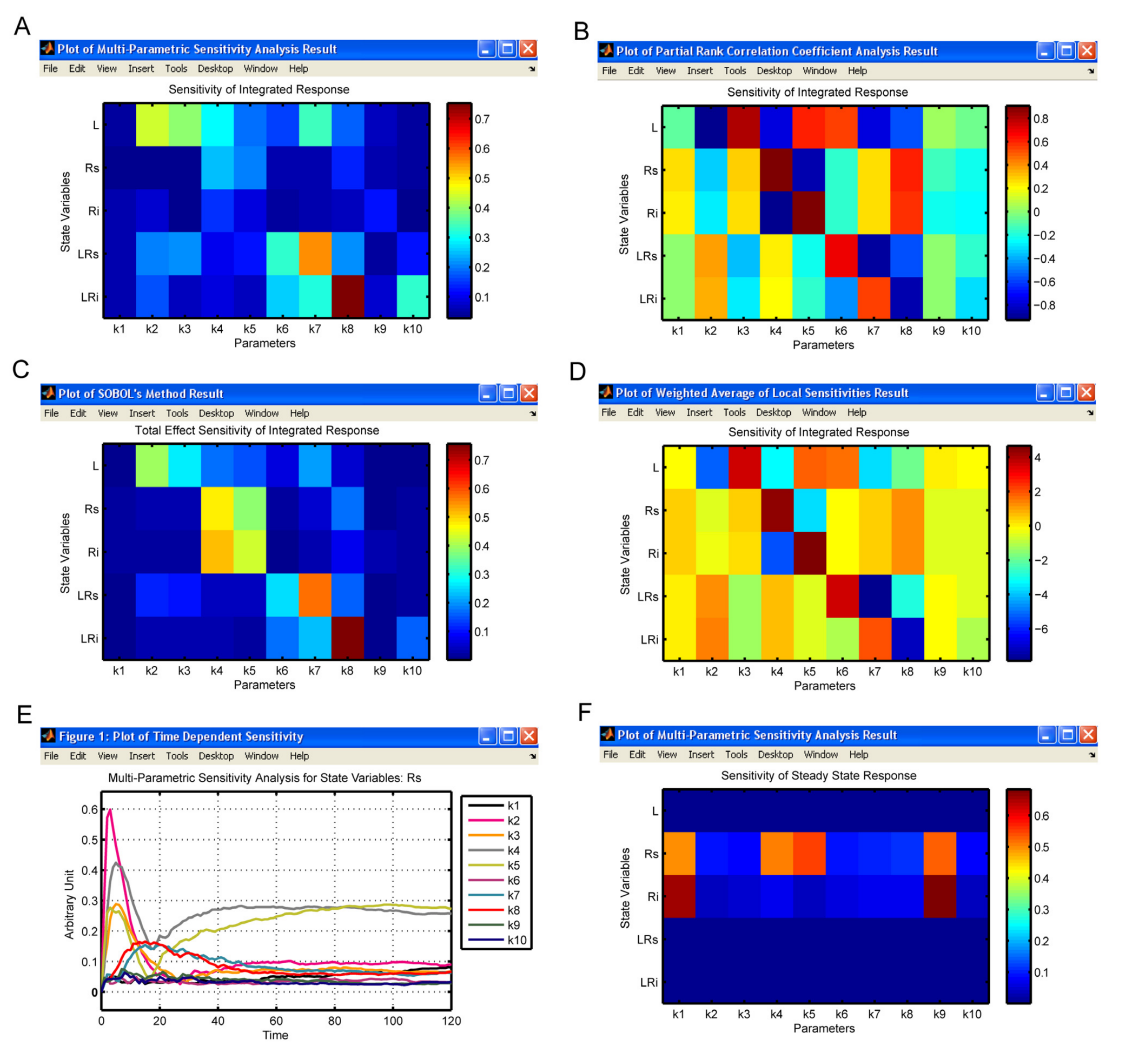
**Global sensitivity analysis in SBML-SAT**. Results of different types of global sensitivity analysis for the receptor trafficking model. (A) MPSA analysis, (B) PRCC analysis, (C) SOBOL's total effect sensitivity analysis, and (D) WALS analysis of integrated response. (E) MPSA analysis of the time dependent response. (F) MPSA analysis of the steady state response.

The results of global sensitivity analysis of the integrated response of the state variables in the receptor trafficking model using all four different methods are shown in Figure 6A–D. The exact values of the sensitivity indices obtained by different methods are not comparable because of their distinct definitions. However, the ranks or relative importance of the parameters to the model output are similar among different global sensitivity analysis methods. The results suggest that the rates of ligand-receptor complexes formation (parameters *k*_2 _and *k*_3_) are very important to the integrated response of ligand concentration (*L*). In contrast, the integrated response of ligand-receptor complexes (*LRs *and *LRi*) are shown to be mainly affected by the rates of the internalization, recycling and dephosphorylation of the occupied receptors (parameters *k*_6_, *k*_7 _and *k*_8_). The MPSA global sensitivity analysis result of the time dependent response (Figure 6E) indicates that *k*_2 _is the key regulator for *R*_*s *_behavior at the early stage (before 20 minutes), but its effect is reduced significantly at a later stage. Upon further analysis, the MPSA global sensitivity analysis of the steady state response (Figure 6F) shows that the steady state of *R*_*s *_is not very sensitive to *k*_2_.

### Sample steady state analysis

A steady state analysis of a user loaded SBML model simply requires to select such analysis from the icons or pull down menu. SBML-SAT initially tries to algebraically solve the system of ODEs for equilibrium solutions. If that fails, the model is simulated over an extended time period to approach the stable steady state related to the initial conditions provided.

**Figure 7:**
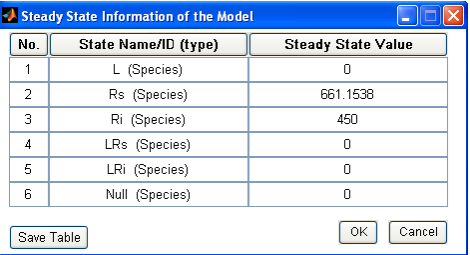
**Steady state analysis in SBML-SAT**. Steady state analysis of the receptor trafficking network model in SBML-SAT.

The results of the steady state analysis of the model of receptor trafficking network are provided in Figure 7. At steady state, all the ligand molecules are taken up by the receptors and eventually degraded, while the internalized and surface receptors that remain unbounded by ligand reach non-zero equilibriums. This information helps to interpret the steady-state global sensitivity analysis results shown in Figure 6F.

### Sample robustness analysis

To conduct a robustness analysis, the user

• loads the SBML model,

• sets the time course,

• chooses the parameter(s),

• defines the variation range of the parameter(s), and

• selects the objects (ODE variables or reaction rates) and the model output operation for the analysis and eventually

• runs the analysis

**Figure 8:**
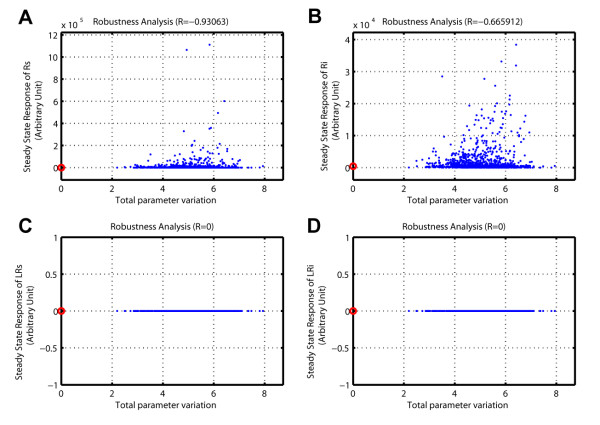
**Robustness analysis in SBML-SAT**. Robustness analysis of the steady state response of the receptor trafficking model against simultaneous variations of the parameter values. The red circles correspond to the reference model output. The blue points correspond to the model outputs under perturbed parameter values. (Specifications for SBML-SAT, "Model Output": "Steady State Response"). (A-B) Robustness of unbound receptor steady state concentration. (C-D) Robustness of ligand bound receptor steady state concentration.

Figure 8 shows the result of robustness analysis of the receptor trafficking model. The steady state concentrations of different forms of receptors are plotted as a function of the total parameter variation (TPV) and the quantitative robustness metric is provided in the subplot title. The results indicate that the steady state concentrations of unbound receptors are less robust to parameter perturbations than the internalized unbound receptor concentration. Not surprisingly, the ligand-bound receptors' concentrations are very robust to the parameter perturbations since their steady state solutions are zero.

## Discussion and conclusion

Currently, a SBML model editor module is not available in SBML-SAT. Fortunately, many existing free software packages such as CellDesigner, SBMLeditor and COPASI, share a common functionality for constructing and editing SBML models. The users can easily generate their models with these free software packages and then run a variety of analyses in SBML-SAT by importing the model in SBML format. Although SBML-SAT doesn't provide a SBML editor for model construction, it provides a convenient track for modifying the initial conditions of the state variables and parameter values in the model. Moreover, delay differential equation models are not supported in SBML-SAT, as in most existing software systems. In practice, delay differential equations can be solved in approximation by converting to ordinary differential equations using the linear chain transformation [41]. Therefore, users can still apply SBML-SAT to their delay differential equation models.

There are more than 120 SBML-supporting software packages for kinetic analysis of biological models and this number continues to grow. However, a powerful, flexible and broadly applicable software package for global sensitivity analysis and robustness analysis has been lacking. In reality, it is difficult and time consuming to implement different sensitivity analysis algorithms especially the global sensitivity analysis methods. Here we introduced, a free Matlab-based software tool, SBML-SAT, for both local and global sensitivity analysis of SBML models. With a user-friendly graphic interface, this tool allows the user to run sensitivity analysis, steady state analysis and robustness analysis for a variety of model outputs. Models involving events are also supported in SBML-SAT. Furthermore, created in Matlab, the most popular software used in the community of systems biology [42], SBML-SAT has a good cross-compatibility with different platforms. Taken all together, we can expect that SBML-SAT will have a broad applicability among systems biologists.

## Availability and requirements

**Project name: **SBML-SAT: A Systems Biology Markup Language (SBML) based Sensitivity Analysis Tool

**Project homepage: **

**Operating system(s): **Windows, Linux, Mac

**Programming language:** Matlab

**Other requirements: **SBMLToolbox, SUNDIALS TB

**License:** none

**Any restrictions to use by non-academics:** none

## Abbreviations

SBML: Systems Biology Markup Language; SBML-SAT: Systems Biology Markup Language based Sensitivity Analysis Tool; MPSA: Multi-Parametric Sensitivity Analysis; PRCC: Partial Rank Correlation Coefficient; WALS: Weighted Average of Local Sensitivities; GUI: Graphic User Interface; LSE: Least Squares Error; TPV: Total Parameter Variation

## Authors' contributions

ZZ proposed the project, designed the GUI interface and wrote all the source code of the software. AR and YZ contributed some algorithms for global sensitivity analysis methods. ZZ, YZ, AR and EK wrote the manuscript and tested the software. All authors have read and approved the final manuscript.
